# AGING AND UTILITARIAN EMOTIONS FOR SOLVING SOCIAL PROBLEMS

**DOI:** 10.1093/geroni/igae098.1994

**Published:** 2024-12-31

**Authors:** Abigail Behrend, Eric Allard, Jennifer Stanley

**Affiliations:** Cleveland State University, Cleveland, Ohio, United States; Cleveland State University, Cleveland, Ohio, United States; University of Akron, Akron, Ohio, United States

## Abstract

Literature suggests a preference for pro-hedonic orientations in older adulthood (Carstensen et al., 2006). Although this is consistent with Socioemotional Selectivity Theory (Carstensen et al., 1999), evidence suggests contra-hedonic motives are viable, particularly in interpersonal scenarios (Allard et al., 2021). We aimed to determine whether young and older adults consider specific emotions useful for accomplishing socially-relevant goals. In an online study, 112 young adults (18-35 years; 29.5% men; 68.8% White) and 72 older adults (64-83 years; 25% men; 93.1% White) viewed 8 interpersonal scenarios with associated social goals. Participants rated how much they would like to engage in emotion-inducing activities on a scale of 1 (not at all) to 7 (extremely) to prepare for each interpersonal interaction. A 4 Goal (Confront, Approach, Avoid, Help-seeking) x 4 Emotion (Happy, Afraid, Angry, Sad) x 2 Age Group (young, old) repeated measures ANOVA revealed a significant Goal x Emotion interaction F (3.82, 695.86) = 210.633 p <.001. Follow-up analyses showed all predicted emotions were endorsed within their intended goal condition (e.g., Approach-Happy, p <.001), with the exception of sadness within the “Help-seeking” condition (p <.001). This partially supports our hypothesis. These results provide additional evidence for age-similarity in utilitarian emotional endorsement. Future research should investigate whether such age similarity remains when experiencing emotions to achieve utilitarian goals.

